# A Group Videogame-Based Physical Activity Program Improves Walking Speed in Older Adults Living With a Serious Mental Illness

**DOI:** 10.1093/geroni/igac049

**Published:** 2022-08-11

**Authors:** Heather Leutwyler, Erin Hubbard, Bruce Cooper

**Affiliations:** Department of Physiological Nursing, University of California, San Francisco, San Francisco, California, USA; Department of Physiological Nursing, University of California, San Francisco, San Francisco, California, USA; Department of Physiological Nursing, University of California, San Francisco, San Francisco, California, USA

**Keywords:** Health disparities, Mental health, Nursing, Physical activity

## Abstract

**Background and Objectives:**

One of the most overlooked populations in our society and in health care are middle-aged and older adults living with a serious mental illness (SMI) despite the growing numbers of this population. Health care communities, including both inpatient and outpatient mental health programs, have a responsibility to provide care that nurtures clients’ mental as well as physical health needs. Providing accessible and engaging physical activity programs is an excellent way to provide this type of holistic care. The purpose of this article is to describe the impact of a pilot videogame-based physical activity program on walking speed in older adults with SMI.

**Research Design and Methods:**

A one-group pretest post-test pilot study was conducted with a sample of 52 older adults with SMI recruited from community-based mental health programs. Participants played an active videogame (using the Kinect for Xbox 360 game system; Microsoft, Redmond, WA) for 50-minute group sessions 3 times a week for 10 weeks. Walking speed was assessed with the timed 3-m walk from the Short Physical Performance Battery at enrollment, 5 weeks, and 10 weeks.

**Results:**

Participants achieved statistically significant improvement in walking speed (0.10 m/s, bias-corrected confidence interval Lower Limit (LL) 0.04, Upper Limit (UL) 0.15) over a 10-week period. This change represents a clinically and statistically (*p* ≤ .05) significant improvement in walking speed. Best estimates for clinically meaningful changes in walking speed are 0.05 m/s for a small change and 0.10 m/s for a substantial change.

**Discussion and Implications:**

Results suggest that engagement in a group videogame-based physical activity program has the potential to improve walking speed in older adults with SMI. In turn, walking speed is an important indicator of premature mortality and cardiorespiratory fitness.

**Translational Significance:** As we design interventions, we need to consider whether the intervention will be affordable, easy to acquire, and easy to implement so as not to further heighten a health inequity. Our videogame-based physical activity program is an example of an affordable intervention that can be nimbly offered in a variety of treatment settings. Our activity is an excellent intervention for inpatient and community-based mental health care environments to provide a low-cost and engaging physical activity within a supportive group setting.

One of the most overlooked populations in our society and in health care are middle-aged and older adults living with a serious mental illness (SMI) despite the growing numbers of this population. Persons in this segment of the population tend to have both acute and chronic medical conditions in addition to disabilities that are attributed to medical and psychiatric origins (1,2). Health care communities, including both inpatient and outpatient mental health programs, have a responsibility to provide care that nurtures clients’ mental and physical health needs. Providing opportunities to engage in physical activity is one way that mental health programs can offer this type of holistic care.

Adults living with an SMI, such as schizophrenia, experience higher rates of morbidity and mortality than the general population and die on average 8–23 years sooner than a person not living with an SMI (2,3). Data suggest that younger people with schizophrenia tend to have poor mobility (4) and their mobility may resemble that of someone 10–20 years older (5). Based on the documented premature mortality, in addition to higher rates of comorbidities prior to reaching the traditional age of older adulthood (ie, 65), many researchers and clinicians conceptualize older adulthood for people living with SMI as starting around 55 years (1,6).

Effective and easily accessible physical activity programs would help to reduce the disability associated with SMI and mitigate some of the other factors that contribute to poor physical health. Research has documented that middle-aged and older adults with SMI need access to engaging physical activity programs and that the lack of available physical activity opportunities contributes to poor health outcomes (7–9). Ample research shows that physical activity promotes better mental and physical health outcomes (10,11). Even short periods of low-intensity physical activity can positively influence mental and physical health (12). Given the multitude of benefits of physical activity, understanding the optimal ways of creating and offering physical activity programs tailored to the needs of people living with an SMI is important.

In a Cochrane review (10), the efficacy of 3 physical activity Randomized Control Trials (RCTs) (ie, walking, combination of walking and jogging, or combination of weight training and aerobic training) in people with schizophrenia (one form of SMI) was evaluated. The authors concluded that the impact of these programs on health outcomes was mixed. One study provided evidence of improved fitness, and all 3 studies showed psychological benefits (eg, less-severe psychiatric symptoms) from physical activity. However, RCTs that compared physical activity interventions with standard care failed to demonstrate changes in other health outcomes. A meta-analysis of 11 RCTs of exercise interventions in patients with schizophrenia was conducted after the Cochrane review (13). The meta-analysis showed similar results. Significant improvements in mental health were observed from interventions that used some form of aerobic exercise (eg, cycling, walking, or playing soccer). Increased adherence was seen in group-based programs over solitary training programs. Six of the RCTs evaluated in the meta-analysis were group-based. Examples of group-based programs from the meta-analysis were soccer and group aerobic programs. In addition, group-based programs may be of particular importance among middle-aged and older people with SMI as high levels of loneliness and social isolation are documented (9,14). More work is needed to understand what will support increased engagement with physical activity programs and improved outcomes among middle-aged and older adults with SMI. Interventions that are available on-site at a mental health program and offered in group format may have a greater impact on health outcomes.

Videogames with an interface that requires physical exertion to play, such as the Kinect for Xbox 360 game system (Microsoft, Redmond, WA), promote physical activity. The Kinect for XBox 360 system does not require the participant to use a controller. Instead, the participant uses their body to directly control the game. The Kinect sensor utilizes full-body tracking that recognizes the participant’s body and mirrors movements in the game. In 2011, the American Heart Association convened a scientific panel that examined whether videogame-based physical activity improved health-related skills and health behaviors (15). The panel included experts in health care, health promotion, behavioral sciences, technology, and game development. They concluded that videogames make physical activity more appealing and engaging for people of all ages and can increase daily activity levels. Videogame research demonstrated that as people become more involved and successful with active videogames, they developed skills that made it easier for them to engage in activity and reported higher levels of physical and emotional well-being (15).

In this article, we present the results of a 10-week videogame-based physical activity program designed for middle-aged and older adults with SMI. Our program was designed and led by a PhD-prepared advanced practice nurse. In our program, 3 times a week for 10 weeks, participants attended 50-minute active group video game sessions using the Kinect for Xbox 360 game system. One of the aims of the program was to determine preliminary efficacy on walking speed. Walking speed is an important indicator of premature mortality and cardiorespiratory fitness (16). Improved walking speed may promote optimal physical health and allow middle-aged and older adults with SMI to maintain independence in their daily life. Additional aims of the study previously reported were to evaluate feasibility and impact on psychiatric symptoms (17). In this article, we focus on the impact our program had on aspects of mobility.

## Method

The 10-week study was designed as a pretest post-test with no control group. This pilot work is intended to inform a larger RCT. Institutional review board approval was obtained from the sponsoring university’s human subjects committee. Anonymity and confidentiality were maintained according to the guidelines set forth by the sponsoring university’s human subjects committee.

Study participants were recruited from the community, direct contact with potential subjects (who previously gave consent to be contacted for future research), and from an outpatient mental health clinic and a transitional residential facility. All potential participants who self-referred or were referred to or recruited by the Principal Investigator (PI) or research assistant (RA) were assessed for the eligibility criteria that consisted of speaking English, 45 years old or older, have a diagnosis of SMI (eg, schizophrenia, schizoaffective disorder, bipolar disorder, psychotic disorder, anxiety disorder, Post Traumatic Stress Disorder (PTSD), or major depression), or have symptoms that indicate risk for psychosis (ie, auditory or visual hallucinations, delusions, sudden decline in self-care). All participants passed a capacity to consent test based on comprehension of the consent form and gave informed consent. The capacity to consent assessment included asking a series of questions which included: What is the purpose of this study? Do you have to take part in the study or is it alright to say no? Participants were excluded if they had known medical conditions or other physical problems that required special attention to engage in an exercise program (eg, prior myocardial infarction, uncontrolled hypertension, history of angioplasty, history of angina, or use of nitroglycerin to treat angina).

For 10 weeks, participants played an active video game using the Kinect for Xbox 360 game system (Microsoft, Redmond, WA) in 50-minute group sessions, 3 times a week. Participants used their body to directly control the game via full-body tracking sensors that mirror movements rather than using a remote control. About 6 feet of free space between the participant and the XBox Kinect sensor was required. The group sessions took place in a multipurpose room at a transitional residential facility or an outpatient clinic. The use of easily available, off-the-shelf video games made it possible for older adults with SMI to participate in physical activities in a group setting on-site at a mental health facility.

Study staff educated participants on warning signs of overexertion (eg, shortness of breath, dizziness) and were instructed to stop exercising if these warning signs occurred. The PI and/or staff were present during all sessions to assist with game set up and to monitor participants. Each 10-week session started with *Bowling* from the Kinect Sports game DVD. Participants were given the option to choose from 10 games at the following sessions, with all participants eventually playing the same 10 games. These game options included *Kinect Sports* (bowling, baseball, golf, tennis, and skiing), *Kinect Dance Central 2*, *Kinect Adventures (River Rush and 20,000 Leaks)*, and *Kinect Your Shape Fitness Evolved (Tai-Chi and Body Focus Workouts)*. One session a week was dedicated to games that promoted coordination and balance, a second session on games that promoted aerobic activity, and the third session on games that promoted strength and flexibility. Participants began the 10-week sessions with single-player turns, so they could learn how to play the games. Some multiplayer games, such as skiing, allowed for groups of up to 6 players to play at the same time. Single-player sessions were still conducted in a group format, such as with bowling. One player bowls at a time, yet the game can be competed as a group. As the participants became more adept at the games, multiplayer games were encouraged, so participants could play for longer periods of time. Group size was capped at 6 players to allow for each player to have an adequate amount of time per turn. Participants were remunerated with 5 dollars at the end of each session for the time needed to complete the sessions and assessments. Upon completion of each session, players rated their perceived exertion with the Borg perceived exertion (BPE) scale (18). The BPE rating was used to help tailor the sessions to each players desired exercise intensity. The PhD-prepared advanced practice nurse monitored intervention fidelity and participant safety.

### Measures

Assessments were completed prior to starting the videogame-based physical activity program and after 5 and 10 weeks of the program. Participants completed questionnaires and participated in assessments about clinical and sociodemographic information. Study staff measured the outcomes and were not blinded. This represents a study limitation yet was necessary as part of a small pilot feasibility study.

Participants’ overall mobility was assessed primarily with the Short Physical Performance Battery (SPPB) (19). The SPPB is a valid and reliable test for quantifying function and mobility and is useful in following the change in mobility over time (20,21). The test takes about 10–15 minutes, requires no special equipment, and can be easily administered at the recruitment sites. The test consists of timed standing balance (side-by-side stand, semi-tandem stand, tandem stand), timed 3-m walk, and a timed test of 5 repetitions of rising from a chair and sitting down. Each of these subtests has a possible score ranging from 0 to 4 and is summed to generate a total score ranging from 0 (worst possible performance) to 12 (best possible performance). The 3-m gait speed and chair stand times are recorded in seconds. We used the timed 3-m walk test as a measure of walking speed over a short distance (19). Best estimates of meaningful clinical change for walking speed are 0.05 m/s for small meaningful clinical change and 0.10 m/s for substantial change (20).

In addition, the 6-minute walk test (6MW) (22) was performed indoors, along a long, flat, straight, enclosed corridor. The walking course was 30 m in length. Participants were instructed to walk back and forth on the course for 6 minutes or as long as possible. Participants were told the object is to walk as far as possible but instructed not to run or jog. A change of 14.0–30.5 m may be clinically important across multiple patient groups (23).

### Analysis

Multilevel linear regression models were used to test change across time for walking speed (24,25). A benefit of the multilevel regression approach over more traditional repeated-measures analysis of variance (RMANOVA) that is often used for this analysis is that multilevel regression models can be estimated when predictors are continuous and categorical (as expected for RMANOVA). The effects of predictors at baseline (enrollment assessment) and the effects of predictors of the change trajectory can be estimated with multilevel regression models. Furthermore, multilevel regression can provide unbiased estimates of change or of the effect of predictors on change whether some assessments of the outcome are missing or participants drop out from the study. The use of full information maximum likelihood (26) with the expectation–maximization algorithm makes this estimation possible (27).

This method provides unbiased parameter estimates if the missingness is ignorable. For example, if participants only provide data at the initial assessment, their data contribute to the estimation of the intercept (eg, mean at baseline) and intercept variance. Participants’ information will contribute to the analysis as many times as they provide data. Any missing data that did occur are assumed to be missing at random (one type of “ignorable”) (26,28). The missing at random assumption is reasonable for the outcomes of interest because it is unlikely that participants did not provide assessments of the measures because of the values that would have been reported had they been assessed.

Distributions of the outcome measures were non-normal (ie, walking speed), and so the general linear model assumption of normal errors was not justified (24,25). Therefore, and in order to allow interpretation of effects on the original scales for walking speed, SPPB total, and 6MW, estimation was carried out with a bootstrap in order to draw inferences about statistical significance. The bootstrap may be carried out in a few ways: with inference based on a *z*-test using bootstrapped standard errors assuming normality, percentile-based confidence intervals, or nonparametric bias-corrected confidence intervals (BC CI). Our analyses presented here employed the nonparametric bootstrap in order to obtain nonparametric bias-corrected bootstrapped confidence intervals using 5 000 repetitions. In using bootstrapped confidence intervals to draw conclusions about statistical significance for our quantitative outcome, a regression coefficient or other effect was determined to be significant if zero was not in the interval. Our analyses were carried out with Stata/SE Release (16, 29), using a 2-sided α of .05.

## Results

Fifty-two participants were included in the analyses. As Table 1 shows, the majority of participants were male. The average age for the overall sample was 59.2 (49–71; 5.3). The majority of participants were current (*n* = 27) or past smokers (*n* = 16). Twenty-nine percent of participants completed at least 3/4 of the sessions and 62% of participants completed at least 1/2 of the 30 sessions. Three participants had perfect attendance.

**Table 1.** Characteristics of Participants (*N = 5*2)

Characteristic	% (*n*)
Male	61.5 (32)
Female	38.5 (20)
White	50 (26)
Black/African American	25 (13)
Latinx	1.9 (1)
Native American	1.9 (1)
Asian	11.5 (6)
Other	9.6 (5)
First language English	88.7 (47)
Past substance use disorder	58 (30)
Schizophrenia	44.2 (23)
Schizoaffective	5.8 (3)
Major depression	21.2 (11)
Anxiety	1.9 (1)
Bipolar disorder	9.6 (5)
Psychosis (not otherwise specified)	7.7 (4)
Posttraumatic stress disorder	9.6 (5)
Residence	
Apartment	9.6 (5)
Board and care	15.4 (8)
Unhoused	3.8 (2)
Psychiatric facility	5.8 (3)
Transitional housing	65.4 (34)

**Figure 1.**
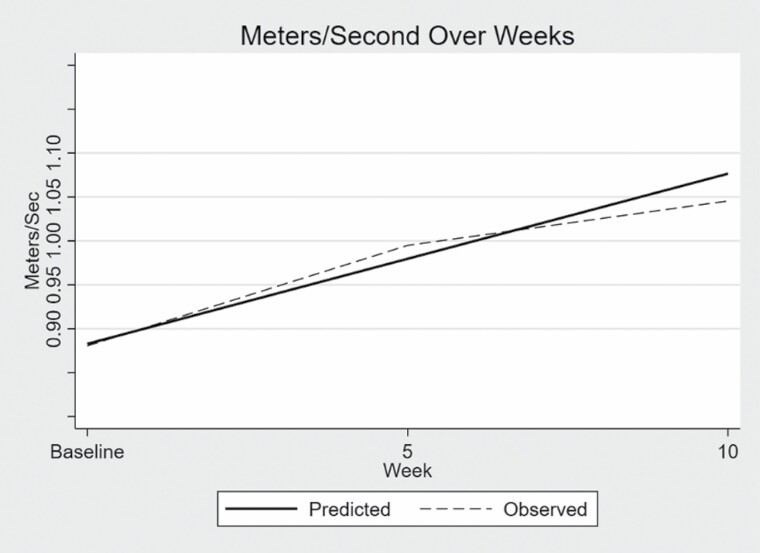
Walking speed.

**Figure 2.**
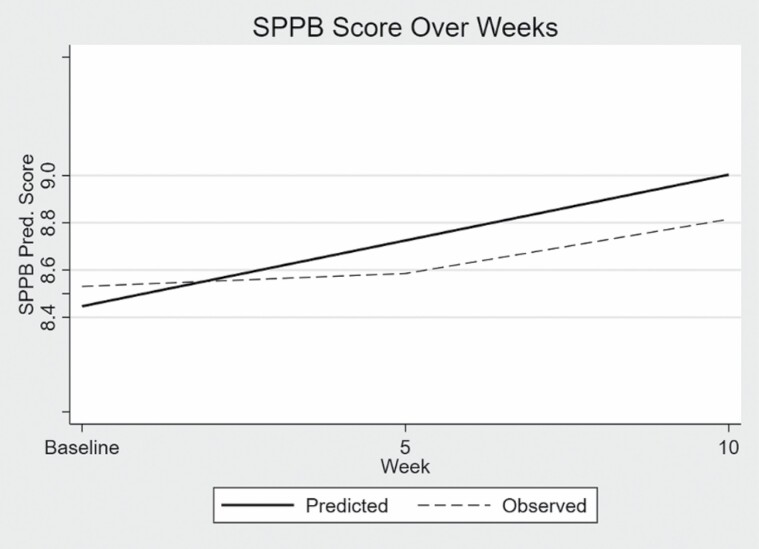
Total Short Physical Performance Battery (SPPB) Score.

Results of the multilevel linear regression are given in Table 2 and illustrated in Figure 1. The change in walking speed as measured by the timed 3-m walk in the SPPB was found to be significant with a 95% confidence interval. The predicted mean score for walking speed from the linear analysis for the sample at enrollment was 0.88 m/s. For each additional 5 weeks of engaging in the physical activity program, walking speed increased on average 0.10 m/s (BC CI LL = 0.04, UL = 0.15) over the entire 10-week study period as illustrated in Figure 1. The increase of 0.10 m/s represents a substantial clinically meaningful change. The predicted mean score for total SPPB score from the linear analysis for the sample at enrollment was 8.45 points. For each additional 5 weeks of engaging in the physical activity program, total SPPB score increased on average 0.06 points (BC CI LL = −0.01, UL = 0.12) over the entire 10-week study period as illustrated in Figure 2. The increase was not clinically or statistically significant.

**Table 2.** Change in Walking Speed on 3-m Course Over a 10-wk Physical Activity Program

Walking Speed (m/s)	Estimate	95% CI	
		Lower Bound	Upper Bound
Initial score	0.88	0.83	0.94
Coefficient	0.10	0.04	0.15

*Notes:* Lower and upper limits for the nonparametric bootstrapped bias-corrected confidence interval (5 000 repetitions): If zero is in the interval, the coefficient is not significant (2-sided α = .05).

**Figure 3.**
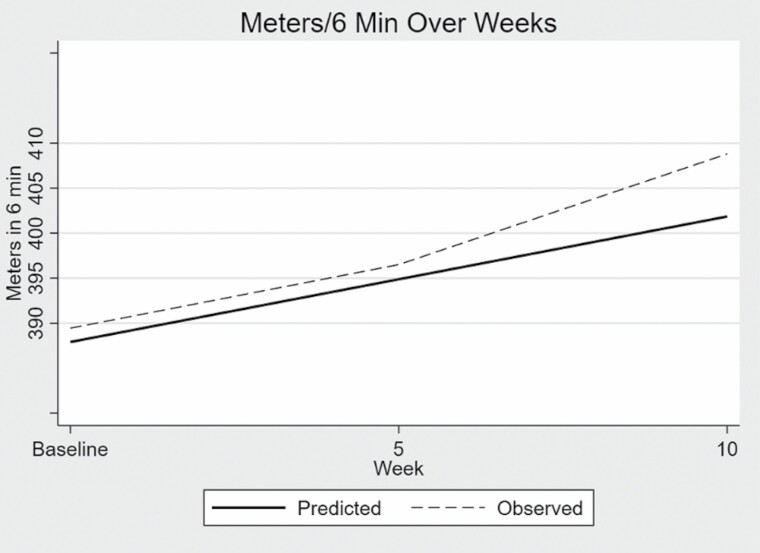
Six-minute walk.

The predicted mean score for 6MW from the linear analysis for the sample at enrollment was 388 m. For each additional 5 weeks of engaging in the physical activity program, meters walked in the 6MW test increased on average 1.40 m (BC CI LL = −1.63, UL = 5.70) over the entire 10-week study period as illustrated in Figure 3. The increase of 1.40 m was not clinically or statistically significant.

## Discussion

Our most important finding was that walking speed on a 3-m test changed over a 10-week physical activity program. Furthermore, our program was engaging, accessible, and introduced participants to activities they can continue beyond the intervention. We adapted the environment where people received care to create more opportunities for physical activity. Creating these types of environmental supports and changes is a necessary step in providing holistic care for people living with an SMI.

A body of physical activity research designed for people with SMI shows a similar impact on walking speed. For example, in a study by Battaglia et al. (30), 18 male participants with a diagnosis of schizophrenia were randomized to one of 2 groups: a trained (TG) or a control group (CG). For 12 weeks, the TG (*n* = 10) was trained with 2 soccer training sessions per week. One of the outcome measures was a 30-m sprint test and slalom test running with a ball. The TG improved significantly on a 30-m sprint test and slalom test running with a ball from baseline to post-test when compared with CG. Battaglia et al. also found the social aspect to be an important feature of their soccer program, attributing some of the success to team activities such as the opportunity for social interaction, sharing leisure activities, competition, and encouraging teammates. We also showed that the social nature of the groups was one of the reasons people were looking forward to returning to each game session (31).

Heggulund et al. (32) conducted a study with 25 female participants from an inpatient setting with a diagnosis of schizophrenia, schizoaffective disorder, or delusional disorder. Participants were allocated to either high aerobic intensity training (HIT; *n* = 12) or playing computer games (*n* = 7). Participants engaged in the programs 3 days per week for 8 weeks. HIT included 4 × 4 minute intervals with 3-minute break periods. Mechanical efficiency of walking (defined as the percentage of work input that is converted into work output) improved by 12% in the HIT group and the improvement was significantly more than the CG group (*p* = .031). Like our study, a change in walking outcomes was found over a relatively short period of time.

Browne et al. (33) conducted the Physical Activity Can Enhance Life (PACE-Life) study. PACE-Life is a multicomponent theory-based walking intervention. Walking groups were sponsored twice per week in the surrounding area of the community mental health center. Seventeen participants with a schizophrenia spectrum disorder diagnosis enrolled and the sample was primarily male (62.50%) and on average 38.2 years old (*SD* = 11.7). The investigators found a large effect size (ES) increase in cardiorespiratory fitness as measured by the 6MW test from baseline to midpoint (ES = 1.17) and baseline to post-test (ES = 1.22). Out of the 13 participants with complete data, 8 at midpoint and 10 at post-test achieved the prespecified threshold for achieving clinically significant improvement (>50-m increase). Similar to our study, the program was group-based and it was offered multiple times per week. Our results stand out from these studies because we include a population that is often excluded from research in schizophrenia (ie, older adults).

### Limitations

We are limited by our small sample size and the lack of statistical or clinical significance on walking speed over a longer distance and SPPB total score. In addition, the lack of a control group limits inferences about our findings. However, we expect with a larger sample to find a more reliable estimate of walking speeds. In addition, the effect we found at the current sample size reduces concerns about sampling error.

## Conclusion

The group-based nature of our videogame-based program addresses one fundamental cause of socioeconomic inequalities in health and mortality, namely lack of social support. As Link and Phelan (34) discussed, putting a focus on fundamental causes of poor health, such as lack of social support, is one way to contextualize our health interventions. Phelan et al. (35) indicate that interventions that seek to change individual risk profiles should first identify factors that put people at risk for risks. Lack of social support may contribute to the more distal causes of poor health in schizophrenia and perpetuate some of the comorbidities experienced. Creating group-based physical activity programs that encourage group play allows for social interactions that can be built during the program and may persist long after the program ends.

Phelan et al. (35) also stated that even if we become more creative in developing contextually based interventions that provide an entire population with health benefits, addressing many health problems will still require individual resources and action. As we design interventions, we need to consider whether the intervention will be affordable, easy to disseminate, and easy to use, so as not to further heighten a health inequity. Our videogame-based physical activity program is an example of an affordable intervention that can be nimbly offered in a variety of treatment settings. Our program is one way to modify mental health care environments, from inpatient to community-based, to provide one avenue to engage in physical activity within a supportive group setting.
